# Progress in Psychological Science. The Importance of Informed Ignorance and Curiosity-Driven Questions

**DOI:** 10.1007/s12124-020-09538-z

**Published:** 2020-05-25

**Authors:** Lucas B Mazur

**Affiliations:** 1 Jagiellonian University, Krakow, Poland; 2 Sigmund Freud University, Berlin, Germany

**Keywords:** Science, Progress, Questions, Theory, Philosophy of science, Ignorance

## Abstract

In recent decades we have seen an exponential growth in the amount of data gathered within psychological research without a corresponding growth of theory that could meaningfully organize these research findings. For this reason, considerable attention today is given to discussions of such broader, higher-order concepts as theory and paradigm. However, another area important to consider is the nature of the questions psychologists are asking. Key to any discussion about the scientific status of psychology or about progress in the field (scientific or otherwise) is the nature of the questions that inspire psychological research. Psychologists concerned about scientific progress and the growth of theory in the field would be well served by more robust conversations about the nature of the questions being asked. Honest, curiosity-driven questions—questions that admit to our ignorance and that express an active and optimistic yearning for what we do not yet know—can help to propel psychology forward in a manner similar to the development of theory or paradigm. However, existing as it does in the “twilight zone” between the natural sciences and the humanities, psychology is fertile ground for questions of wide-ranging natures, and thus the nature of progress in the field can be variously understood, not all of which will be “scientific.” Recent psychological research in three areas (cognition, memory, and disorders/differences of sex development) are discussed as examples of how curiosity-driven questions being asked from a position of informed ignorance can lead to progress in the field.

## Introduction

It is “the right question, asked the right way, rather than the accumulation of more data, that allows a field to progress.” (Firestein 2012, p. 98)

In recent decades we have seen an exponential growth in the amount of data gathered within psychological research. Study after study is conducted to test various hypotheses, and yet in psychology at large there is little consensus regarding the core concepts in use, and there is a general absence of broader theories or overarching paradigms that would allow for the meaningful organization of research findings (Valsiner 2017; Zagaria et al. 2020). For this reason, considerable attention today is given to discussions of such broader or higher-order concepts as theory and paradigm, and rightly so. Within such discussions, some psychologists have argued that the field would be well served by adopting an inclusive, pluralistic, “meta-theory,” such as evolutionary psychology (Zagaria et al. 2020). The main aim of the current piece is to suggest that, before looking for unifying (“scientific”) theories, psychologists interested in the scientific progress of the field should look into the nature of the questions being asked and the degree to which those questions open the door to the possibility of scientific progress in the first place. We will examine some characteristics of the kinds of questions that allow for the possibility of scientific progress, and we will briefly look at some areas in psychology in which we see such questions being asked. Much psychological work—valid and valuable psychological work—is not scientific, and much good work would be difficult to link with any notion of progress in the field, scientific or otherwise. For many psychologists, and within a considerable amount of psychological work, the “soft” status of the field is not a concern. Thus, before we can assess the potential merits of various meta-theories, it would seem to be important that we first ask if we are generally studying psychology in a manner that is suggestive of a collective, scientific undertaking, and thus one in which inclusive, pluralistic, meta-theories would potentially be of use. By examining the nature of our questions, we can explore the degree to which the practices of psychologists point towards the possibility of various versions of scientific progress in the field, or perhaps in different, non-scientific directions.

As assessed along a number of metrics (e.g., the theories-to-laws ratio, the rate of consultation between researchers in the field, citation frequency of new researchers, citation concentration), psychology is considered a “soft science” relative to the natural sciences (Zagaria et al. 2020). In addition to these factors, the soft status of psychology also arises from the nature of the questions that give impetus to psychological research. In as far as those questions are curiosity-driven—which is to say that they profess an “informed ignorance” and an honest curiosity about the world—they afford us a way to meaningfully assess progress (scientific or otherwise). Honest, curiosity-driven questions are both a confession of ignorance and a profession of openness to the as-yet-unknown that lies beyond. “Thoroughly conscious ignorance is the prelude to every real advance in science” (James Clerk Maxwell quoted in Firestein 2012, p. 7).

While reflecting on the nature of particular questions or of questions in general can feel philosophically naïve or overwhelmingly complicated in turn, the matter is worth raising here in this brief piece as in practice psychologists rarely explicitly engage in such reflection. Psychologists rarely, *in practice*, publicly profess ignorance, and do so even more rarely as a badge of honor, as an assertion of hope, as a rallying cry. Rather than reflecting on the sense of wonder that can appear in our ignorance, psychologists often in effect seem to want themselves and others to wonder at what is already known. Rather than exploring the unknown, psychologists focus more on the methods we use to get to know it, and the theories we hope to developed to know it better. By focusing exceedingly on issues of method, methodology, hypothesis, theory, or goals, psychologists often lose sight of the object of study, and in the process the curiosity driving our questions about the world often becomes of secondary importance. Unless we are pushed forward by our ignorance and curiosity, and allow ourselves to be pulled forward by what we find outside of ourselves in response, the scientific status of psychology will remain standing on what Zagaria et al. (2020) call “clay feet.” The question of psychology’s scientific status and the possibilities of scientific progress in the field pertain not so much to whether or not psychology can stand on its own, as whether or not it has anywhere to go.

### Progress Arising from Informed Ignorance

The notion of progress in science is both exceedingly popular and particularly slippery (e.g., Debrock and Hulswit 1994; Laudan 1978). Our current understanding of scientific progress developed in the West over the past several centuries, and has been understood in several different ways over that time (for a particularly helpful explication of that history, see Harrison 2015). In as far as we can speak of science centuries ago, the idea of scientific progress was initially understood as the perennially new, individual-level cultivation of internal virtue with the assistance of a semiotically pregnant world (as in Aquinas’s understanding of *scientia*). More recently, it has come to be seen as a linear, collective-level accumulation of external, objective data from a mechanistic world. The philosopher Charles Taylor (2007) refers to these changes as part of the shift from an “enchanted” to a “disenchanted” world. Over the course of this transition, science came to be understood as an endeavor divorced from other areas of knowledge, such as those found in the humanities and the arts (Daston and Galison 2007). For many, science has come to be seen not just as a method, but also a philosophy; a shift that others find problematic (e.g., Sheen 2019). Psychology emerged as an independent field in the nineteenth century during a particularly intense period of this transition, and debates regarding psychology’s academic allegiances were there from the very beginning (Rzepa and Dobroczyński 2019; Valsiner 2012). Much like the historical development of nationally-conscious languages whose promotors made decisions regarding phonetics, grammar, and lexicon so as to differentiate their languages from those of neighboring peoples (Snyder 2003), psychologists worked very hard to distance the field from those with which it was the most similar, particularly philosophy (e.g., as seen in the work of Gustav Fechner on “psychophysics,” in the famous laboratory of Wilhelm Wundt, or in the intellectual development and interesting career path of Władysław Heinrich between psychology, philosophy, and pedagogy; Danziger 1990; Rzepa and Dobroczyński 2019). Many early psychologists fought to steer psychology in the direction of the natural sciences, and in doing so, largely adopted a “disenchanted” understanding of progress.

Closely related to the notion of scientific progress, and similarly complex, is an activity that also appears at first glance to be patently simple, namely, asking questions (Firestein 2012). The nature of the questions we ask about the world gives shape to the nature of our scientific theories. Similarly, our broader understandings of the world give shape to the questions we ask. Thus, questions can be thought of as containing both deductive and inductive elements (and constituting a “chicken or egg” dilemma). Amidst the intellectual battles accompanying the historical “disenchantment” of the world, nineteenth century social scientists were well aware of how the questions we pose are expressions not only of what we don’t know, but also of our intellectual allegiances to existing schools of thought (e.g., Sheen 2019). For example, Auguste Comte (1798–1857) wanted the new field of “social physics” (later “sociology”) to be a purely “scientific” endeavor, free from metaphysical or philosophical musings. In its allegiance to empirical science and its rejection of metaphysics, Comte’s positivism finds expression in the questions he believed social scientists should ask and therefore “takes the terrestrial horizon as its boundary, without prejudice to what might lie beyond it. Abandoning too high a level of speculation, it retired to lowlier positions of more immediate interest” (de Lubac 1995, p. 159). Thus, in the writings of many early social scientists—academics who were acutely aware that the intellectual allegiances and academic independence of their newly emerging fields were in doubt—we see a clear awareness that questions come in all shapes and sizes, and that not all questions are equal regarding the direction in which they propel us (moral and value judgments aside) (Sehon 2005). They were aware that it is not only important to ask questions, but also that it is important to ask questions about the questions we are asking.

#### Curiosity-Driven Questions

It is not easy to ask meaningful questions, but doing so is of fundamental importance for the advance of any scientific field. Neurobiologist, Stuart Firestein (2012), has argued that within the natural sciences it is “the right question, asked the right way, rather than the accumulation of more data, that allows a field to progress” (p. 98). In as far as we see its status as a “soft science” as problematic, we can say that psychology, on the whole, is struggling to ask meaningful questions *in the right way,* that is, in a way that would allow for scientific progress to be made. In as far as psychology can be considered a science or even an academic pursuit, it is important that psychologists reflect on the questions being asked in the field. Asking honest questions indicates an awareness of our ignorance, as well as a curiosity about what lies beneath.

We have been long aware of differences in quality between the questions we ask. For example, C. S. Peirce (1958) argued that real questions arise from genuine, “living” doubt, not what he calls “paper doubts,” that is, questions that arise from the compulsive need to ask questions—as often found among academics—rather than from honest curiosity and an awareness of one’s own ignorance. “According to Peirce, the doubt that brings forth inquiry must be genuine. It is not sufficient to say or write that one doubts; ‘paper doubt’ does not amount to legitimate disbelief” (Bergman 2009, p. 16). With this distinction in mind, we can also see that there is a subtle, but fundamental, difference between curiosity-driven questions and hypothesis testing. While not mutually exclusive, and ideally complementary, in practice hypothesis testing often overshadows genuine questions in psychological research. Not only is there the overwhelming pressure in academia to publish “significant findings,” but those findings are generally expected to confirm the initial hypotheses. Within published psychological research there are few surprises. This applies to both quantitative and qualitative research (even in the absence of explicit hypotheses, as is often the case of the latter). In this climate, unsurprisingly, psychologists often become cheerleaders for their own hypotheses (or general expectations). Additional theoretical, political, professional, and social biases also abound (Campbell and Manning 2018; Duarte et al. 2015; Ferguson and Heene 2012; Gerber et al. 2001), making the majority of research presented in published articles or at conferences thoroughly predictable. While hypothesis testing can be helpful to the extent that it contains within itself the mechanisms for assessing “significance” and thereby for meaningfully judging outcomes, it is unhelpful to the extent that we favor an outcome before the fact. Thoroughly conscious ignorance requires the meaningful assessment of research outcomes, as afforded by hypothesis testing, but also an openness to, and genuine curiosity of, those outcomes. If the outcomes are essentially contained within the hypotheses, and known in advance, the process is not future oriented, and no progress can be made. Having lost sight of the tension within every honest question between what is known and what is unknown, hypotheses in psychology are often, in practice, rhetorical questions.

Another relatively small, but not unimportant, indicator of this can be found in the questions that appear in the limitations section at the end of research articles (rather than lying at the core of the work). What is more, the limitations or suggestions for future research are often actually backhanded suggestions in support of the given claims (e.g., usually by suggesting extensions or replications). Such practices in effect anchor psychological questions to the past and to the already-known, rather than projecting them into the future and the as-yet-unknown. In as far as we root for a hypothesis, and do so at the expense of genuine, curiosity-driven questions, we run the risk of silencing the data and ignoring the subject. This can also happen when our constructs are too rigidly defined, indicating that a lack of universal agreement about even core constructs in the field is not necessarily a bad thing, that is, as long as this flexibility allows us to turn meaningfully to the data, or rather, to the subject under study. In fact, intentionally loosening up on widely held definitions can shift priority from the researcher and the past (the known), to the subject and the future (the as-yet-unknown), and yet, “That principle is one that is difficult for many scientists to swallow, because it relaxes control, gets the experimenter out of the driver’s seat, and leaves it up to the subjects […] to produce the results” (Firestein 2012, p. 97). The generative quality of honest questions, as well as the thorough excitement and deep discomfort that such inquiry can cause, is wonderfully expressed in the following statement made by Robert Boyle (1627–1691):

“…an Inquisitive Naturalist finds his work to increase daily upon his hands, and the event of his past Toils, whether it be good or bad, does but engage him into new ones, either to free himself from his scruples, or improve his successes. So, that, though the pleasure of making Physical Discoveries, is, in it self consider’d, very great; yet this does not a little impair it, that the same attempts which afford that delight, do so frequently beget both anxious Doubts, and a disquieting Curiosity.” (quoted in Hunter 2000, p. 13).

#### Criteria for Judging the Data

Since at least the times of ancient Greece we have been wrestling with the “dilemma of dualism,” i.e., how it is that the seeker of knowledge can come to know that which is unknown (Debrock and Hulswit 1994). How can one recognize that which one has never yet seen? While not resolving this age-old riddle, we can recognize that asking questions presupposes the ability to assess the meaningfulness of what one hears in response. It presupposes the (at least partial) intelligibility of what follows the question; it presumes the ability to see in a response an answer. In this sense, questions contain broader conceptualizations that meaningfully combine individual data. While grounded in what we already know (or think we know), asking curiosity-driven questions is also essentially about the future and our confident movement into that future (Firestein 2012; Valsiner 2017). Honest, curiosity-driven questions—questions that admit to our ignorance and that express an active yearning for what we do not yet know—can help to propel psychology forward in a manner similar to the development of theory or paradigm. The advances that we see in the natural sciences have come from the ability of scientists in those fields to ask honest, curiosity-driven questions and to meaningfully judge the various responses they get from the natural world in reply. Answered questions inspire new questions, more data are gathered, theories and paradigms emerge to meaningfully organize and compare the “answers” we have found, and the march of progress is set afoot. Such progress is seen relatively rarely in psychology, at least in part, because of the nature of the questions being asked. The range of questions asked by psychologists reflects the wide range of epistemological and ontological positions professed in the field. For example, broadly speaking, there are psychologists who understand psychology to be an empirical, experimental science (e.g., “all psychology must be based on experiment, and that it is quite improper to set aside an area of study labeled ‘experimental’ as if to suggest that other areas of psychology exist which are not experimental”; Bugelski 1951), just as there are those who believe it can never be an empirical science (e.g., “an objective, accumulative, empirical and theoretical science of psychology is an impossible project”; Smedslund 2016, p. 185). These two broad camps exist, and will continue to exist, convinced of the value of their enterprise (Mazur and Watzlawik 2016). Gustaw Ichheiser (1897–1969) saw this fundamental tension not as an impasse, but as an opportunity: “[S]ocial scientists should, in my opinion, not aspire to be as ‘scientific’ and ‘exact’ as physicists or mathematicians, but should cheerfully accept the fact that what they are doing belongs to the twilight zone between science and literature” (cited in Rudmin et al. 1987, p. 171).

Psychology belongs to this twilight zone in part because the body of criteria psychologists use for perceiving data as answers to their questions is broad, inclusive, and often shifting—and arguably more so than in other fields. While literary scholars *generally* do not use “scientific” criteria to study literature, and physicists *generally* do not use the judgement criteria of literature within physics, psychologists more readily and more often shift between the criteria associated with one or the other of what C. P. Snow (1959) called “the two cultures” (i.e., the sciences and the humanities). While many, such as Ichheiser, see this as a strength of psychology, it is also arguably responsible for a considerable degree of confusion (Kagan 2009). Although the general conceptual separation of the “two cultures” is a relatively recent historical development (Harrison 2015), and one that many have argued is not as clear-cut even today as may appear (Gould 2003; McAllister 1996; Sullivan 1933), in as far as we give credence to this and similar distinctions between fields of study, we ought to take seriously the differences in judgment criteria both between and within fields. This is particularly important within psychology precisely because psychologists often oscillate between various judgment criteria, none of which has primacy within the field. Judging the meaningfulness of data involves the tension between the restrictiveness of judgment criteria that focus our vision and an openness of those criteria so as to not lose sight of their limitations relative to the object of study, however, this essential tension can lose its “bite” if one can in effect shape that balance at will.

As suggested by Ichheiser and others, flexibility in method and methodology within psychology can be a strength, that is, as long as it opens up new avenues for fruitfully studying the subject. After all, polyvalence is understood to be part of our psychological lives (Boesch 1991). However, there is a double-sided risk that comes with this flexibility. On the one hand, we can switch between judgment criteria too often and too easily, thereby ultimately devaluing what the subject says to us in our research; after all, criteria for determining “significance” (quantitative or otherwise) are ultimately intended to serve our ability to perceive the subject in ways that would otherwise be unavailable to us. On the other hand, we can swing too far in the other direction, becoming too wedded to any one judgment criteria over others, thereby undercutting the richness of our conceptual toolkit and, more importantly, of the subject. When facing the complexities of the world, judgment criteria are helpful precisely because they allow us to perceive the world, to perceive the subject, in ways that would otherwise have escaped our awareness. Balance in the use of judgment criteria saves us from both “trivial order” and “barbaric vagueness”; from the “extremes of premature closure and narrow-mindedness on the one hand, and interminable indecision and ‘broadmindedness’ on the other” (Aeschliman 1983, p. 69–71). To simplify matters somewhat, we see this balancing act illustrated in the tension that often emerges in psychology between qualitatively-minded researchers and quantitatively-minded researchers. Qualitatively-minded researchers often criticize quantitative, experimental research as being overly restrictive or even closed to the subject (“forcing the subject into the empirical methods”), while quantitatively-minded researchers often criticize qualitative research as using weak or “wishy-washy” criteria for evaluating the subject. By holding onto our techniques too tightly, we ignore other options, overlook the limits of method, and ultimately restrict potential discoveries. By letting go too much of such criteria, we ignore the power of those techniques and thus we deny the subject the chance to speak to us through them. By either overly restricting or overly expanding our criteria we in effect lose sight of the subject.

### Examples of Curiosity-Driven Questions

To suggest that we reflect more on the nature of the questions we ask, or to suggest the value of curiosity-driven questions for psychological science, is not to suggest the value of any concrete question(s) in particular. What is more, given the nature of curiosity-driven questions arising from informed ignorance, it is impossible to identify such questions on face value (that is, on the basis of any particular wording). In other words, while we have been referring to this as a “question,” or rather a type of question, it is in reality more of an approach or practice. Just as the question “Why am I sick?” can be scientific, moral, rhetorical, etc. (Sehon 2005), any particular motivating question posed by psychologists can be of various natures. Similarly, as curiosity-driven questions are defined by their relation to informed ignorance and their receptiveness to the subject, they necessarily extend in time and space beyond what we usually think of as the question itself. Thus, any example of such a question would need to explore the foundations provided by informed ignorance and the manner in which the question indicates a responsiveness to the subject (and the “data”). For this reason it is perhaps more accurate to think of them as research practices or processes.

An example of such a process can be seen in psychological research on consciousness. The notion of consciousness is of fundamental importance within psychology, but also in broader, non-academic discussions. It is also one of those often used, but incredibly “fuzzy,” concepts within the field (Zagaria et al. 2020). One area of research within this general topic concerns whether non-human animals have what we call consciousness. As we remain somewhat unsure about just exactly what this term means, it is particularly difficult to look for it empirically. However, as researchers have learned to let go of their “human biases” and listened to their non-human subjects, they have expanded their view on how consciousness might “look” in non-human animals and how we might study it there (Firestein 2012). For example, rather than expecting consciousness to appear like a conversation between two adult humans, researchers have been making strong claims for the presence of consciousness in a wide range of animals (e.g., Plotnik et al. 2010). In a related line of research, work on theory of mind (ToM) continues to produce new tests to identify the appearance and development of such elements of consciousness in children at various ages (Jakubowska and Bialecka-Pikul 2020; Wellman et al. 2001). This has required researchers to in effect stop thinking like adult scientists and to start thinking like younger and younger children. By listening to how children see and interact with the world, psychologists have come to better understand the development of the perception of mental states, both one’s own and those of others. In the case of both children and animals, psychologists have been asking questions out of a position of informed ignorance and they have been listening to their subjects. As a result, not only is it fairly safe to say that progress has been made, but new questions have emerged, and continue to emerge, as a result. Such research also builds the collective body of knowledge on the subject, a hallmark of modern science (Harrison 2015), and such curiosity towards the subject makes the research of others relevant, especially more recent research—another hallmark of “hard” science (Zagaria et al. 2020).

Another area of research that constitutes a nice example of the kinds of questions here under consideration concerns the nature of memory. Psychologists have been interested in the nature of memory since the very beginnings of the field. When looking across the history of memory research, hiccups and oddities aside, one would be hard-pressed to defend the position that progress has not been made, even “scientific” progress. Across a wide range of research methodologies and methods, a diverse group of researchers have expanded our knowledge of the complex, plastic, and dynamic nature of memory (e.g., Lamprecht and LeDoux 2004). Researchers have been open to their subject(s) and as a result, have come to suggest radical changes to our conceptualizations of memory, or rather, memories (Bourtchouladze 2004). That the issue of memory continues to puzzle and fascinate researchers indicates not the scientific failure of the field, but the expansive, generative nature of honest inquiry.

Another example can be seen in research over the past several decades on gender within cases of “Disorders/Differences of Sex Development” (DSD; formerly called intersex, hermaphroditism, pseudohermaphroditism, sex errors of the body, or ambiguous genitalia). DSD should not be confused with what is known as “gender dysphoria” (APA 2013), that is, cases in which a person believes their gender identity to not match their biological sex or the gender to which they were assigned. DSDs have been defined as “congenital conditions in which development of chromosomal, gonadal, or anatomical sex is atypical” (Lee et al. 2006). Cases of DSD challenge traditional thinking regarding gender identity development by showing that such development is complex, involving numerous prenatal and postnatal factors. For example, someone could be born with 46,XY chromosomes (i.e., a male karyotype), internal testes and no internal female reproductive organs, but female appearing external genitalia. Such a person is likely to be assigned a female at birth and/or thought of as female by their family, thereby beginning a process of female gender identity development which is discordant with their chromosomal and gonadal status. What is more, in the presence of complete androgen insensitivity syndrome (CAIS), such an individual may come to further develop an external female phenotype (e.g., developing breasts). Similarly, there is a large and growing body of research on “classical” congenital adrenal hyperplasia (CAH) in individuals with a 46,XX karyotype, wherein these individuals show marked hormone abnormalities (e.g., related to androgens), which result in various forms and degrees of “masculinization” (Meyer-Bahlburg 2014). DSDs raise fascinating questions regarding the nature of gender, not to mentioning the countless other areas of life connected thereto (e.g., sexual attraction, sexual functioning, reproduction, various non-sexual behavioral patterns, self-image, cognitive functioning, and mental health).

Scientific knowledge regarding various DSDs has increased over the past several decades (e.g., Lee et al. 2006; Lee et al. 2016), as has our understanding of the biological, psychosocial, and cultural factors that contribute to what we generally call gender, including “gendered behavior” and “gender identity” (Meyer-Bahlburg 2014). By better understanding DSDs in particular, we are able to better understand gender in general. What is more, not only does there remain a great wealth of ignorance in this area from which curiosity-driven questions can arise, but the more “informed” that ignorance becomes, the more curiosity-driven questions emerge. However, psychological research in this areas is not only concerned with scientific progress, as generally understood, but also with issues related to such matters as quality of life, quality of relationships, purpose, sense of self, and personal growth, issues which may or may not be what we would often consider “scientific” considerations. What is more, there is also a considerable and growing amount of activism related to DSDs. Progress can mean different things to different people working in this field. In other words, in cases of DSD (as elsewhere in psychology, including work on consciousness and memory), the questions psychologists ask are not *necessarily* those that lead to *scientific* progress. In fact, psychological work in this area is often clearly non-scientific. There also remains considerable disagreement regarding just what the science is supposed to say beyond the realms of method. Taken together, this is an example of the “twilight zone” of which Ichheiser spoke and with which he was both comfortable and confident.

Within research on all three of the areas discussed above we not only find strong claims that scientific progress has been made, but also strong claims that a considerable portion of psychological work is *not* scientific. What is more, there are also disagreements about the ways in which, or the degree to which, science can, or should, inform other areas of our lives. Again, this is not a judgement regarding the value of scientific or non-scientific work, but merely to point out the existence of differing undertakings within the field. One such division can be found between the assertion of the known that we see in advocacy and the assertion of the unknown that we see in science. Much has been written about the complex relationship between science and advocacy, and it is a topic that extends well beyond the scope of the current piece. However, for practical purposes it is worth reflecting on a basic difference between science and advocacy, even if that involves somewhat stereotyped and simplified images of both. Broadly speaking, while advocacy involves the promotion of what we know, or think we know, science promotes our ignorance and directs us towards what we do not yet know—and it does so again and again. Advocacy can stymie scientific progress by restricting what can and cannot be asked, by pushing an agenda regardless of what research might be produced—in other words, by restricting our access to the subject. However, advocacy can also encourage researchers to pose new, curiosity-driven questions that are responsive to the subject. Just as advocacy can inspire or restrict science, science can both support and/or hamper advocacy. There is no inherent moral value to this conflict, as science and advocacy can work both wonders and horrors. While the intertwining natures of science and advocacy is certainly much more complex than this, this distinction remains of considerable practical use, especially when looking at the questions that drive psychological work.

The point here is that if we listen closely to the questions psychologists ask today we will often hear many different theoretical positions and numerous fundamentally different understandings of progress (e.g., see the questions posed by 33 “influential psychologists” in APA 2018). In that regard, such distinctions as the science/advocacy difference (among others) remain of practical utility, at least in as far as psychologists are concerned with the *scientific* development of the field, or with progress in the field (however defined). As discussed above, within discussions of the *scientific* status of psychology it is worth reflecting on the assumptions contained in the questions posed, the degree to which they are future-oriented, and the degree to which they are responsive to, and receptive of, the subject. At the same time, not all meaningful and valuable questions need to lay the foundations for scientific progress, or any kind of progress for that matter (e.g., Sehon 2005). Nevertheless, questions remain of fundamental importance for science and for the notion of progress, scientific or otherwise.

## Conclusion

Ideally, theory both grounds us in what we know and propels us into the unknown. In light of this, it is certainly worth reflecting on, and attempting to readjust, the current imbalance in psychology between data and theory. At the same time, it is worth examining another important part of the research process, namely, the nature of our questions. What is it that captures our genuine wonder? Where does the recognition of our ignorance simultaneously evoke a belief that we can overcome it? It is there that we find the liminal space between the known and the as-yet-unknown; it is there that we can being to meaningfully trace potential progress and to identify the nature of that progress (not all of which will be “scientific” as currently understood). In our concern for the progress of “psychological science,” it is worth reflecting on the potentially progressive quality of our questions. Do they reflect an honest curiosity about the world? Are they boldly expressive and humbly receptive? To the extent that they balance between the known and the unknown, and between the “trivial order” and the “barbaric vagueness” of the given field, it is important that we recognize that not all questions are equal. Questions come in a wide range of types and they serve all sorts of ends, not all of which bespeak the possibility of scientific progress as currently understood. This is not in itself problematic, far from it. Yet, for psychologists concerned with the scientific status of the field or with the possibility of its progress as an empirical science, it is worth asking if our questions provide fertile ground for the notion of such progress in the first place.
